# Clinical and genetic characteristics of 36 children with Joubert syndrome

**DOI:** 10.3389/fped.2023.1102639

**Published:** 2023-07-21

**Authors:** Yan Dong, Ke Zhang, He Yao, Tianming Jia, Jun Wang, Dengna Zhu, Falin Xu, Meiying Cheng, Shichao Zhao, Xiaoyi Shi

**Affiliations:** ^1^ Department of Pediatrics, Third Affiliated Hospital of Zhengzhou University, Zhengzhou, China; ^2^ Henan Key Laboratory of Child Brain Injury, Institute of Neuroscience and Third Affiliated Hospital of Zhengzhou University, Zhengzhou, China; ^3^ Academy of Medical Sciences, Zhengzhou University, Zhengzhou, China; ^4^ Department of Children Rehabilitation, Third Affiliated Hospital of Zhengzhou University, Zhengzhou, China; ^5^ Department of Radiology, Third Affiliated Hospital of Zhengzhou University, Zhengzhou, China; ^6^ Department of Pediatric Development and Behavior, Third Affiliated Hospital of Zhengzhou University, Zhengzhou, China

**Keywords:** Joubert syndrome, ataxia, hypotonia, genetics, whole exon sequencing, molar tooth sign, bat wing sign

## Abstract

**Background and aims:**

Joubert syndrome (JBTS, OMIM # 213300) is a group of ciliopathies characterized by mid-hindbrain malformation, developmental delay, hypotonia, oculomotor apraxia, and breathing abnormalities. Molar tooth sign in brain imaging is the hallmark for diagnosing JBTS. It is a clinically and genetically heterogeneous disorder involving mutations in more than 40 ciliopathy-related genes. However, long-term follow-up data are scarce, and further research is needed to determine the abundant phenotypes and genetics of this disorder. The study aimed to summarize clinical manifestations, particular appearance on cranial imaging, genetic data, and prognostic features of patients with JBTS.

**Methods:**

A retrospective case review of 36 cases of JBTS from May 1986 to December 2021 was performed. Clinical data of JBTS patients with development retardation and molar tooth sign on cranial imaging as the main features were analyzed. Genetic testing was performed according to consent obtained from patients and their families. The Gesell Developmental Scale was used to evaluate the intelligence level before and after treatment. The children were divided into a purely neurological JBTS (pure JBTS) group and JBTS with multi-organ system involvement group and then followed up every 3–6 months.

**Results:**

We enrolled 18 males and 18 females. Thirty-four (94.44%) cases had developmental delay, one patient (2.78%) had strabismus, and one patient (2.78%) had intermittent dizziness. There was one case co-morbid with Lesch-Nyhan syndrome. Three-quarters of cases had one or more other organ or system involvement, with a greater predilection for vision and hearing impairment. JBTS could also involve the skin. Thirty-one cases (86.11%) showed a typical molar tooth sign, and five cases showed a bat wing sign on cranial imaging. Abnormal video electroencephalogram (VEEG) result was obtained in 7.69% of cases. We found six JBTS-related *novel* gene loci variants: *CPLANE1*: c.4189 + 1G > A, c.3101T > C(p.Ile1034Thr), c.3733T > C (p.Cys1245Arg), c.4080G > A(p.Lys1360=); *RPGRIP1l*: c.1351-11A > G; *CEP120*: c.214 C > T(p.Arg72Cys). The *CHD7* gene may be potentially related to the occurrence of JBTS. Analysis showed that the prognosis of pure JBTS was better than that of JBTS with neurological and non-neurological involvement after the formal rehabilitation treatment (*P* < 0.05). Of the three children with seizures, two cases had epilepsy with a poor prognosis, and another case had breath-holding spells.

**Conclusion:**

Our findings indicate that early cranial imaging is helpful for the etiological diagnosis of children with unexplained developmental delay and multiple malformations. Patients with JBTS may have coexisting skin abnormalities. The *novel* gene loci of *CPLANE1*, *RPGRIP1l*, and *CEP120* were associated with JBTS in our study and provided significant information to enrich the related genetic data. Future works investigating several aspects of the association between *CHD7* gene and JBTS merit further investigation. The prognosis of children with pure JBTS is better than that of children with JBTS with non-neurological involvement.

## 1. Introduction

Joubert syndrome (JBTS, OMIM # 213300) was first reported by Joubert et al. in 1969 (1) and is a form of the neurodevelopmental disorder with autosomal recessive or X-linked recessive inheritance. Over recent years, autosomal dominant inheritance with partial penetrance has been suggested (2). The main manifestations of JBTS are ataxia, hypotonia, *oculomotor apraxia*, abnormal respiratory rhythm, cognitive impairment, and development retardation. Some of these symptoms are evident from the neonatal period (3, 4). In addition, JBTS may be associated with abnormalities of other organs, such as the retina, kidney, liver, and bones (1). Typical imaging features of JBTS are hypoplasia or absence of the cerebellar vermis and reduction or absence of the decussation of the superior cerebellar peduncles and corticospinal tract, a condition referred to as a molar tooth sign (MTS). The possibility of JBTS should be considered if a child has unexplained development retardation, multiple malformations, partial or complete loss of the cerebellar vermis, widening of the superior cerebellar peduncles, and enlarged fourth ventricle. The clinical manifestations of the disease vary widely; thus, there is a major difference in the severity of this condition. However, there are few studies on JBTS in large populations, especially in China. Furthermore, there are only a few long-time follow-up studies investigating the development of JBTS.

In this study, we followed up children with JBTS diagnosed and treated in the Third Affiliated Hospital of Zhengzhou University (Zhengzhou, China) since its establishment in May 1986. We summarized clinical characteristics, imaging, genetics, and long-term follow-up data to improve our understanding of the disease.

## 2. Materials and methods

### 2.1. Patients and study design

We selected patients who were diagnosed with JBTS by clinical examinations and cranial imaging and treated them in the outpatient or inpatient department of the Third Affiliated Hospital of Zhengzhou University between May 1986 and December 2021. We collected and analyzed medical records of all the patients with JBTS, including medical history, physical examination, routine blood tests, biochemical and genetic testing, cranial imaging, ultrasonic testing, neurocognitive assessments, and video electroencephalogram (VEEG). Patients were followed up as outpatients or by telephone every 3–6 months to assess height, weight, and cognitive and physical development regularly. We used the Gesell Developmental Schedules (GDS) to evaluate the neurodevelopmental level of children with JBTS, which has been widely used to evaluate the development of children aged from 16 days to 6 years (5, 6).

The diagnosis of JBTS was made according to the imaging diagnostic criteria proposed by Maria et al. (7). The inclusion criteria were as follows: (1) molar tooth sign: a thickened and extended upper cerebellar peduncles, hypoplasia of the cerebellar vermis, and the lateral view of the shape similar to the molars; (2) midline sign: a midline fissure sign refers to line-like low-density shadows and long T1 and T2 signal shadows between the two cerebellar hemispheres; (3) bat wing sign or triangular fourth ventricle: the upper part of the fourth ventricle is shaped similarly to a bat wing, and the middle part is triangular due to hypoplasia of the cerebellar vermis. Patients were excluded if their guardians refused to provide information or cooperate with follow-up or if clinical data were incomplete. During the follow-up, the children were divided into a purely neurological JBTS (pure JBTS) group and a JBTS with multi-organ system involvement group.

This study was reviewed and approved by the ethical standards of the Third Affiliated Hospital of Zhengzhou University's Research Ethics Committees (Zhengzhou, China; reference: 2021-062-01). The written informed consent was waived from the legal guardian/next of kin in accordance with the committees referred to above.

### 2.2. Magnetic resonance imaging (MRI)/computed tomography (CT)

Brain MRI was performed on a Siemens Skyra 3.0 T (Siemens, Germany) superconducting MR scanner with an 8-channel array surface coil. Axial, sagittal, and coronal images were acquired. Brain CT was performed with a Siemens Somatom Definition AS + 64-slice CT with CARE Dose 4D technology (SOMATOM Definition flash, Germany). The images were analyzed by two radiologists with more than 10 years of experience.

### 2.3. Whole-exon sequencing (WES)

We extracted 2 ml of peripheral blood from children and parents and then used the blood genome extraction kit (Kangweishiji, Beijing, China) for DNA extraction. Following the quality control and library establishment, DNA fragments in the target region were enriched, and the whole exon library was constructed. The probes were captured by xGen® Exome Research Panel v2.0 (IDT, Iowa, USA). An MGISEQ-T7 series sequencer (BGI, Shenzhen, China) was used for high-throughput sequencing, and the sequencing process was performed by Beijing Chigene Translational Medicine Research Center (Beijing, China). Following the quality control and using Burrows-Wheeler Aligner software (BWA, version 0.59, http://bio-bwa.sourceforge.net/), the paired-end clean reads were compared to the Ensemble reference genome (GRCh37/hg19). Genome Analysis ToolKit software (GATK v4.0, https://gatk.broadinstitute.org/) was used to filter and screen detected single nucleotide polymorphisms (SNPs) and indels to obtain high-quality and reliable variants. The detected variants were then annotated by an online high-throughput sequencing database, the Mendell Genetic Disease Analysis System (http://www.chigene.org). Data were analyzed in combination with standardized clinical phenotypes, genetic patterns, and public databases. Databases such as dbSNP (https://www.ncbi.nlm.nih.gov/SNP/), 1,000 Genomes (https://www.ncbi.nlm.nih.gov/genome/gdv/browser/genome/), ExAC (http://www.gnomad-sg.org/), and ESP (ttps://evs.gs.washington.edu/EVS/) were then used to detect minimum allele frequencies. Using software for protein structure prediction, including Provean (http://provean.jcvi.org/index.php), SIFT (http://sift-dna.org), PolyPhen22 (http:/genetics.bwh.harvard.edu/pph2/), and Mutationtaster (http://www.mutationtaster.org/), we predicted protein product structure variation. Variants were assessed for pathogenicity according to guidelines provided by the American College of Medical Genetics (ACMG) (8). Suspected pathogenic mutation sites were primed and sequenced using an ABI3730 Automated Sequencer (PE Biosystems, Foster City, CA) to complete Sanger verification.

### 2.4. Follow-up and prognosis evaluation

All 36 children with JBTS were followed up by outpatient or telephone appointments every 3–6 months. We used the Gesell Developmental Scale (GDS) to evaluate the intelligence level before and after treatment (5, 6). The GDS developed by Gesell and Amatruda is a useful tool to determine the integrity and maturity of the nervous system in children (9). The Chinese version of the GDS was revised by the Chinese Pediatric Association in 1986 and has been shown to exhibit strong internal reliability and validity (10). The Chinese version of GDS items is grouped into five neurodevelopmental domains: gross motor, fine motor, adaptability, language, and social behavior. According to Chinese general practice, the development quotient (DQ) scores for the total and five clinical domains were classified into “low (DQ < 70)”, “middle and lower (70 ≤ DQ ≤ 84)”, “medium (85 ≤ DQ ≤ 114)”, “middle and upper (115 ≤ DQ ≤ 129)”, or “high (DQ ≥ 130)” level. The neurodevelopmental delay was diagnosed when an infant's neurodevelopmental status was rated as “middle and lower” and “low” (11). The efficacy of the rehabilitation treatment of the patients was evaluated according to GDS.

### 2.5. Statistical comparisons between groups

All statistical data analyses were performed by using SPSS version 26.0 (IBM Inc., Chicago, IL, USA). Quantitative data of normal distribution were shown as mean ± standard deviation, while the non-normally distributed data were expressed as median (quartile) [*M* (*P25*, *P75*)]. Qualitative data were shown as *n* (%). The chi-square test or Fisher exact probability test was used for quantitative data, and the Student's *t*-test or the Wilcoxon rank sum test was used for qualitative data. A *P*-value ≤ 0.05 was considered statistically significant.

## 3. Results

### 3.1. Clinical data

A total of 36 children were enrolled in this study, including 18 men and 18 women. The age of onset ranged from 5 days to 11 years. One patient was diagnosed by MRI due to dizziness when she was 11 years old; this scenario was markedly different from other children. After removing this patient, the age of onset followed a normal distribution with a mean age of 11.96 ± 9.83 months. Of 36 children, almost 94.44% of patients had a global developmental delay, one case suffered from strabismus, and one case had vertigo. There were 19 cases of hypotonia and two cases of hypertonia. Fifteen cases had ocular diseases, including six cases of nystagmus, six cases of strabismus, three cases of retinal abnormalities, two cases of optic nerve misrouting, two cases of abnormality of the optic nerve, one case of bilateral abnormal optic disc morphology, and four cases had two or three of these ocular abnormalities.

Eight cases were complicated with hearing abnormality, including one case with cochlear malformation and one case with bilateral abnormal vestibular saccule morphology. Abnormal respiratory rhythm occurred in seven cases, including six cases with neonatal respiratory distress and one case with neonatal intermittent apnea. There were seven cases with urinary system involvement, four cases with mildly abnormal renal function, two cases with nephrolithiasis, and one case with abnormal renal collecting system morphology. Skin abnormalities were detected in five cases, presenting as skin rash, allergy, and recurrent skin infections. Abnormal cardiac structure or function occurred in five cases: three cases had abnormal muscle tissue enzyme activity, two cases had an atrial septal defect, and one of the atrial septal defect patients also had left ventricular systolic dysfunction. There were four cases with liver damage: three cases with elevated hepatic transaminase and one case with hepatosplenomegaly.

There were three cases with distinctive facial features, including hypertelorism, wide nasal bridge, and underdeveloped nasolabial fold. Among the three patients with seizures, two patients were diagnosed with epilepsy with tonic-clonic seizures, while Lesch-Nyhan syndrome, hallmarked by hyperuricemia, intellectual disability, and self-mutilation coexisted in one of the patients. The third patient had a focal autonomic seizure with hypoventilation/hyperventilation/altered respiration. One patient had occipital encephalocele. There were two cases of polydactyly, one case with preaxial hand polydactyly, and one case with postaxial foot polydactyly (Supplementary Table S1).

### 3.2. Cranial imaging findings

**Figure 1:**
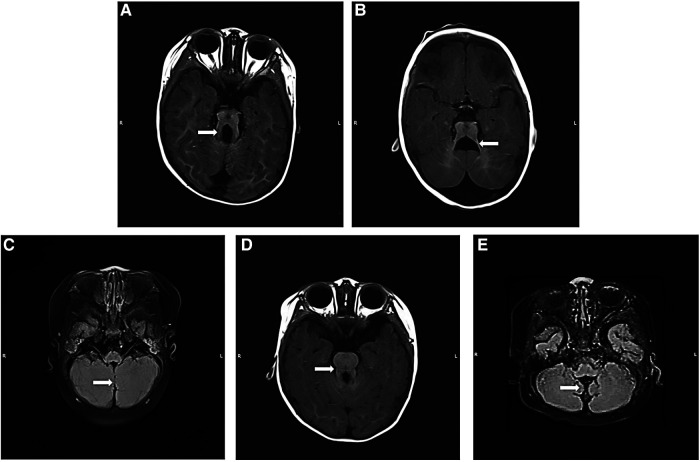
Brain MRI findings of Joubert syndrome children. (**A**) Axial T1WI: “molar tooth sign”; (**B**) axial T1WI: “bat wing sign”; (**C**) T2WI flair: “midline fissure sign”; (**D**) axial T1WI: the typical imaging findings of a patient with *CPLANE1* variant Joubert syndrome showing a “molar tooth sign”; this sign was less obvious in other patients; (**E**) T2WI flair: typical “hypoplasia of the cerebellar vermis”, “morphological abnormality of the fourth ventricle” and “thickened, elongated superior cerebellar peduncles” in the child with *CHD7* gene variant. T1WI, T1 weighted imaging; T2WI, T2 weighted imaging.

Of 36 patients, 35 patients were diagnosed with JBTS by MRI, and one patient was diagnosed by CT. All patients had cerebellar vermis dysplasia (Figure 1). Of these, 19 had a small or thin vermis, 10 had a partial or complete absence of the vermis, and 7 had the division of the vermis. Of these, 31 cases showed a typical molar tooth sign on brain MRI (Figure 1A), 5 cases showed the enlarged fourth ventricle with a bat wing sign (Figure 1B), and 25 patients had both of these abnormalities. In addition, 18 cases showed cerebellar fissures (Figure 1C). Except for typical imaging findings, 11 cases showed enlarged lateral ventricles, three cases were complicated by intracranial cysts, two cases were complicated by periventricular leukomalacia, and one case was complicated by agenesis of the corpus callosum.

### 3.3. Video electroencephalogram findings

A total of 24 patients underwent VEEG examinations. There were two VEEG abnormalities in two epileptic patients. These were characterized by diffuse slow waves with overlapping sharp waves during wakefulness and sleep (Supplementary Table S1).

### 3.4. Whole-exon sequencing

**Figure 2:**
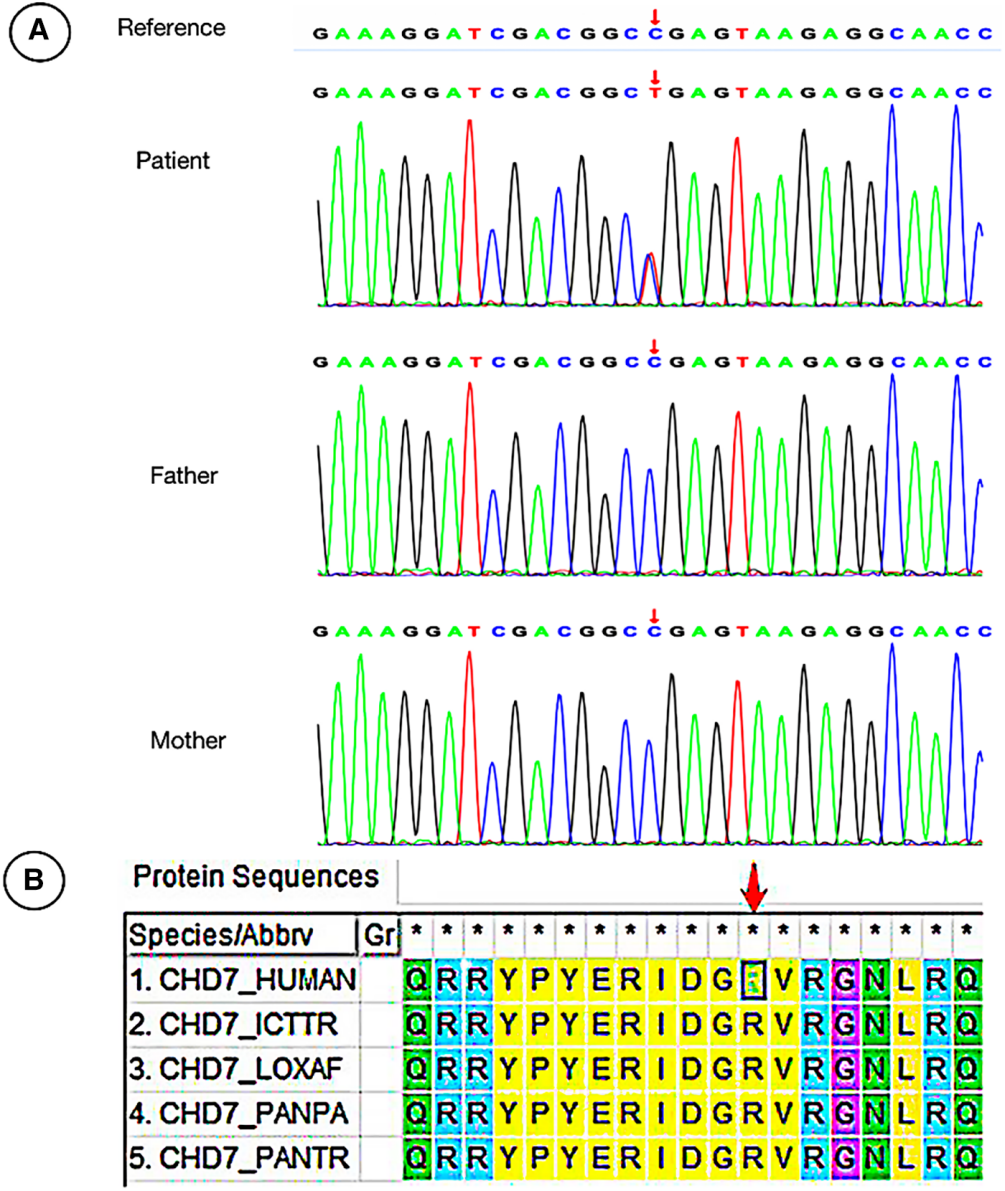
Sanger sequencing of the patient with *CHD7* variation and the evolution analysis of different species. (**A**) The male patient carried a heterozygous missense mutation c.4015C > T (p. Arg1339*) in the *CHD7* gene, while his Proband's parents did not carry the variation. (**B**) Phylogenetic analysis of *CHD7* proteins from different species.

We reviewed clinical data and found that WES was performed in nine cases, including three cases with *CPLANE1* gene variants, two cases with *AHI1* variants, one case with *RPGRIP1l* variant, and one case with *CEP120* variant (Table 1). The remaining patients did not undergo WES for family reasons. Some variants had been reported in JBTS previously, while there were six gene loci in this study that had never been reported related to JBTS before, including *CPLANE1*: c.4189 + 1G > A, c.3101T > C(p.Ile1034Thr), c.3733T > C(p.Cys1245Arg), and c.4080G > A(p.Lys1360=); *RPGRIP1l*: c.1351-11A > G; *CEP120*: c.214 C > T(p.Arg72Cys). Among them, the *RPGRIP1l*: c.1351-11A > G had been reported in a Chinese family diagnosed Meckel syndrome (12). The WES results showed that there was a JBTS patient with *CHD7* variation c.4015C > T(p.Arg1339*) in exon 17 (Table 1, Figure 2). Variations in gene *CHD7* caused the majority of CHARGE syndrome cases (13); however, there are only two studies that identified an association between CHARGE syndrome and JBTS (14, 15), and one of these studies was by our research team. This suggests that the *CHD7* gene may be related to the occurrence of JBTS. Whether *CHD7* is a *novel* gene for JBTS needs further investigation (Figure 2B).

**Table 1:** Gene variants.

Proband	Cases	Genes	Mode of inheritance	Nucleic acid mutations	Amino acid variation	ACMG Classification	Pathogenic factors
1	case 8	*RPGRIP1l*	AR	NM_001127897.3:c.3354G > A	p. Trp1118*	P	PVS1 + PM2 + PP4
				NM_001127897.3:c.1351-11A > G	-	LP	PM2 + PM3 + PP3
2	case 9	*CHD7*	AD	NM_017780:c.4015C > T	p.Arg1339*	P	PVS1 + PS2 + PM2 + PP5 + PP3
3	case 15	*CPLANE1*	AR	NM_023073.3:c.4189 + 1G > A	-	P	PVS1 + PM2 + PP4
				NM_023073.3:c.3101T > C	p.Ile1034Thr	VUS	PM2 + PM3 + PP4
4	case 14	*CPLANE1*	AR	NM_023073:c.7978C > T	p.Arg2660*	P	PVS1 + PM2 + PP5 + PP3
				NM_023073:c.4080G > A	p. Lys1360=	VUS	PM2 + PM3 + PP3
5	case 17	*CPLANE1*	AR	NM_023073.3:c.7243dup	p.Thr2415Asnfs*7	LP	PVS1 + PM2
				NM_023073.3:c.3733T > C	p.Cys1245Arg	VUS	PM2 + PP3
6	case 29	*CEP120*	AR	NM_153223.3:c.1684del	p.Thr562Leufs*4	P	PVS1 + PM2 + PP4
				NM_153223.3:c.214C > T	p.Arg72Cys	LP	PM2 + PM3 + PP3 + PP4
7	case 22	*AHI1*	AR	NM_001134831.2:c.2168G > A	p.Arg723Gln	P	PM2 + PP1 + PP4 + PP3
				NM_001134831.2:c.2174G > A	p.Trp725*	P	PM2 + PP1 + PP4 + PP3
8	case 23	*AHI1*	AR	NM_001134831.2:c.2168G > A	p.Arg723Gln	P	PM2 + PP1 + PP4 + PP3
				NM_001134831.2c.2174G > A	p.Trp725*	P	PM2 + PP1 + PP4 + PP3

AD, autosomal dominant inheritance; AR, autosomal recessive inheritance; LP, likely pathogenic; P, pathogenic; VUS, variant of uncertain significant.

### 3.5. Follow-up

The follow-up period was 34.87 ± 14.33 months. All 36 patients received rehabilitation treatment, including kinesitherapy, physiotherapy and traditional Chinese medicine. It was effective in 20 of these patients; however, 16 cases did not respond to treatment. Of these patients, 12 had a developmental arrest, two had developmental regression, and two patients died (Supplementary Table S1). Following regular rehabilitation, the prognosis of the pure JBTS group was significantly better than that of the other group (*P *< 0.05) (Table 2).

**Table 2:** Comparison of the efficacy of rehabilitation treatment between children with pure JBTS and others.

Groups	Prognosis		Total cases	*P*-value
	Effective and efficient cases	Invalid cases		
Pure JBTS	8	1	9	0.03
Others	12	15	27	

It is a comparison of the efficacy of regular treatment in different groups over 34.87 ± 14.33 years.

Of the three children with seizures, two were diagnosed with epilepsy, and one was diagnosed with breath-holding spells. One of the children with epilepsy died of a serious infection 3 months after diagnosis. Although one of the children was prescribed valproate (30 mg/kg/d) and levetiracetam (60 mg/kg/d), they still experienced recurrent seizures.

## 4. Discussion

Joubert syndrome, first reported by French neurologist Marie Joubert in 1969, is a rare neurodevelopmental disorder with multiple organ system involvement that is associated with cerebellar vermis dysplasia and abnormal morphology of the fourth ventricle (1). The prevalence of JBTS in a population aged 0–19 years is 1.7 per 100 000, and the male-to-female ratio is approximately 3:2 (16). In this study, the male-to-female ratio was 1:1, which may be related to the small sample size, different regions, and ethnic groups.

In this study, JBTS tended to involve the nervous system, vision, and hearing. There was a hitherto unreported clinical case co-morbid with Lesch-Nyhan syndrome in our center. Moreover, JBTS can involve the skin. We collected clinical data and indicated that *CHD7* might be associated with JBTS. Furthermore, early cranial MRI is helpful for the etiological diagnosis of children with unexplained developmental delay and multiple malformations. An aspect neglected was that the ratio of the abnormal VEEG in JBTS without epilepsy was low. Numerous studies have reported clinical, imaging, and molecular genetic findings of JBTS patients (17–19); however, there is still a lack of long-term follow-up and large sample size studies. To the best of our knowledge, this is the first study to comprehensively evaluate the interrelations between pure JBTS and JBTS with non-neurological involvement in China. We further confirmed that patients with pure JBTS obtained better outcomes after regular rehabilitation.

The neurological features of JBTS are hypotonia progressing to ataxia, global developmental delay, oculomotor apraxia, and respiratory abnormalities. JBTS may also be accompanied by different degrees of involvement of other organs, such as the retina, kidney, bones, and liver (3). In addition, JBTS exhibits high levels of clinical and genetic heterogeneity. Some researchers believe that the diagnostic criteria for JBTS are typical imaging features, clinical hypotonia, and developmental delay (3, 20). Other researchers believe that ocular abnormalities and respiratory abnormalities are also involved (21). In the present study, all the findings in the pediatric patients were consistent with an abnormal cranial MRI structure. Our review of clinical data showed that 94.44% of children had developmental retardation, although some children only had dizziness or strabismus. Two cases had normal developmental milestones. Altogether, JBTS is considered to be related to different types of pathogenic genes, loci, variation patterns, and resulting functional changes, although these factors need further investigation.

Previously, JBTS has been divided into eight clinical subtypes according to the presence or absence of extrinsic central nervous system features, namely purely neurological JBTS (pure JBTS), JBTS with ocular involvement (JBTS-O), JBTS with renal involvement (JBTS-R), JBTS with oculorenal involvement (JBTS-OR), JBTS with hepatic involvement (JBTS-H, or COACH syndrome), JBTS with oral-facial-digital involvement (JBTS-OFD, or OFDVI syndrome), JBTS with acrocallosal features, and JBTS with Jeune asphyxiating thoracic dystrophy (3, 22). According to this classification, our study included nine cases of pure JBTS (25.00%). In addition, in 75% of patients, the disease affected one or more organs or systems, and eyes (41.67%) and hearing (22.22%) were frequently involved. Retinal photoreceptors play a key role in the formation of vision, and their outer segments are special sensory cilia capable of participating in the transmission of light (23). Therefore, abnormalities in the structure and function of the cilia caused by genetic variations can lead to the shortening of the outer segments and photoreceptor cell dysfunction, resulting in retinal degeneration. The vestibular and cochlear motor cilia play their function by oscillating (24), and abnormalities in this process can lead to damage to the auditory system (25, 26). In a study by the National Institutes of Health and University of Washington (17, 27), retinal involvement was present in 24%−30% of individuals, although few results have been related to hearing in the study mentioned. Thus, we considered that the reason was related to the early postnatal fundus examination and hearing examination of patients, which improved the detection rate of patients with related system abnormalities in our study.

**Figure 3:**
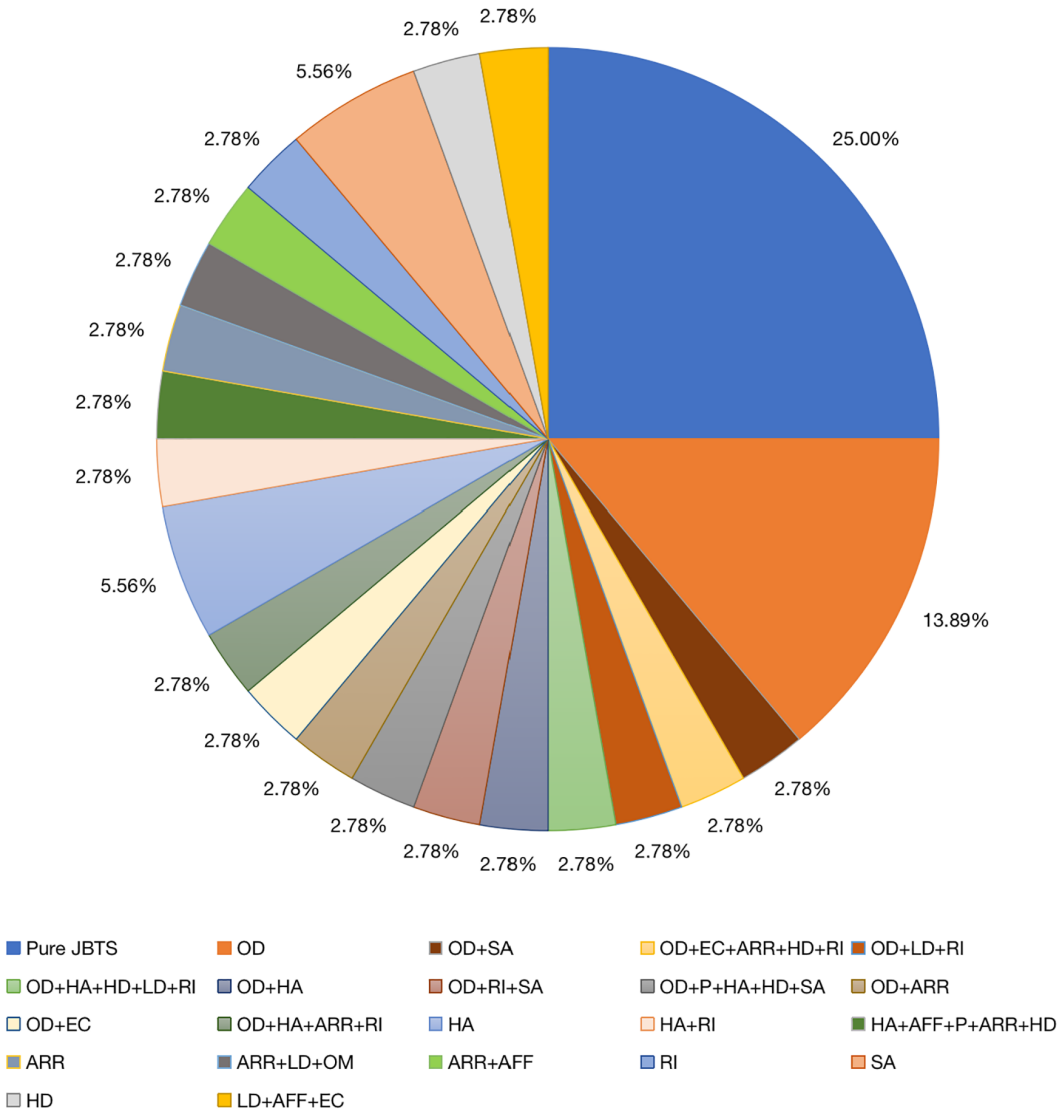
Organ involvement in 36 children. AFF, abnormal facial features; ARR, abnormal respiratory rhythm; EC, epileptic complications; HA, hearing abnormalities; HD, heart disease; LD, liver damage; OD, ocular diseases; OM, occipital encephalocele; P, polydactyly; Pure JBTS, Joubert syndrome involving the central nervous system only; RI, renal injury; SA, skin abnormalities.

In the present study, we found that five children had skin abnormalities and were prone to rash, allergy, and recurrent skin infections. Primary cilia in epidermal cells control cell morphological changes by regulating directional cell division, cell differentiation, and orientation of the hair follicle plane (28). A previous study found that primary cilia are involved in physiological processes such as keratinocyte differentiation, hair formation, and epidermal stress (29). JBTS, as a ciliopathy, is often associated with cilia-related gene mutations. Therefore, we hypothesize that JBTS patients might develop skin abnormalities because of the variants in genes associated with ciliary function. These findings have not been previously reported and, thus, provide further enrichment of the clinical phenotype of JBTS (Figure 3). Other children had liver damage, abnormal urinary systems, distinctive facial features, abnormal cardiac structure or function, and polydactyly, and these findings were consistent with previously published data (30). However, the proportion of affected organs varies among different centers. Considering the genotypes involved, this might be related to the fact that all children included in this study were Asian, or it was limited by the sample size. Because not all patients in our study could be included in the present classification system, further revision of JBTS classification is expected in the future. Among the seven patients with urinary system involvement, two cases with abnormal renal function underwent WES, and *RPGRIP1l* and *CHD7* gene variants were detected. Previous studies have reported that JBTS due to *RPGRIP1l* gene variants are mostly combined with renal involvement (31, 32), our study is consistent with the results. *CHD7* gene variation mainly caused CHARGE syndrome, a single-gene genetic disorder with multiple organ malformations including ocular coloboma, congenital heart defects, choanal atresia, retardation of growth and development, genital hypoplasia, and ear anomalies associated with deafness (33), and rarely be found associated with renal involvement. Although the predictive elements of renal damage such as concentrating power, minirin test were not performed further in our patients, we will follow up them in order to trace and assess the evolution of the renal involvement.

Molar tooth sign is a typical sign of JBTS imaging diagnosis, and other signs include midline fissure, bat wing sign, or triangle sign, which are also typical imaging findings (34). There are many other abnormalities in addition to the manifestations described above. For example, Poretti et al. analyzed 75 cases and found that children with JBTS also showed cranial imaging manifestations such as hippocampal malrotation, dysgenesis of the corpus callosum, absence of the septum pellucidum, occipital encephalocele, and giant ventricles (22). Poretti et al. also reported abnormal neuronal migration, including periventricular nodular gray matter heterotopia, multiple cerebellar gyri malformation, and focal cortical dysplasia (22). In the present study, we identified one patient that had occipital encephalocele. In addition, Enokizono et al. found that patients with *CPLANE1*-variant JBTS could be easily misdiagnosed due to mild changes on imaging. In the presen study, one of the three patients with *CPLANE1* variants had a typical MTS (Figure 1D) (35). Other study have indicated that ultrasound revealing hypoplastic cerebellum and cerebellar vermis of the fetus may also point to JBTS (36). Therefore, this is of great significance for the prenatal diagnosis of JBTS.

The pathogenesis of JBTS is related to the abnormal structure and function of cilia (37). Primary cilia are receptors for mechanical and chemical signals, which play an important role in embryonic development, genetic diseases, and even tumorigenesis (38). Thus far, more than 40 JBTS-related genes have been identified. In one study, researchers performed WES on 100 patients with JBTS from different countries and found that the common variants were *TMEM67*, *CPLANE1*, *CC2D2A*, *CEP290*, *AHI1*, *KIAA0586*, *MKS1*, and *INPP5E* (17). *TMEM237*, *NPHP1*, *RPGRIP1l*, *OFD1*, *SUFU*, and other genes have also been related to JBTS; of these, *OFD1* gene variant has been linked to X-linked recessive inheritance (39). *SUFU* gene variant has been associated with autosomal dominant inheritance, and the remaining gene variants showed autosomal recessive inheritance (2). A total of nine WES cases were analyzed in this study. We found six *novel* variations related to JBTS, including *CPLANE1*: c.4189 + 1G > A, c.3101T > C (p.Ile1034Thr), c.3733T > C (p.Cys1245Arg), and c.4080G > A(p.Lys1360=); *RPGRIP1l*: c.1351-11A > G; *CEP120*: c.214C > T (p.Arg72Cys). There were two splicing variations among them, c.4189 + 1G > A of *CPLANE1* was located in the exon 24, which has not been reported in previous study. However, c.4162C > G of *CPLANE1* has been detected in a Japanese family with JBTS (40); hence, we hypothesize that the alterations cause amino acid variants in nearby regions, resulting in abnormal protein expression. The other splicing variation is *RPGRIP1l*: c.1351-11A > G, which has only been found in a Chinese family with Meckel syndrome (12), but never been reported about this locus and the nearby regions related to JBTS. This locus was predicted by spliceAI to affect splicing, and the mutation was consistent with the phenotype. Therefore, according to the American College of Medical Genetics (ACMG) guidelines, this locus was judged as likely pathogenic. Moreover, splice site mutation effects on the protein deserve more attention and research.

*CPLANE1*, also known as *C5orf42*, causes JBTS type 31. Its mode of inheritance is autosomal recessive, and it was the most common related gene in the study. It is most common in pure JBTS and JBTS with polydactyly; however, there is a low correlation between JBTS and kidney and retinal diseases (41). A Northern European cohort study of JBTS found that *CPLANE1* variants accounted for 12% of all cases of JBTS but predominantly caused pure JBTS (42). Of the three children with *CPLANE1* mutations in our study, two were diagnosed with pure JBTS. This was consistent with previous studies (42). However, one child was diagnosed with epilepsy and Lesch-Nyhan syndrome. Lesch-Nyhan syndrome is a congenital X-linked recessive neurogenetic disorder caused by variation in the hypoxanthine-guanine phosphoribosyl transferase (*HPRT*) gene (43). Aggressive, self-mutilative behavior is probably the most striking aspect of LNS, and in most patients, the hallmark is the loss of tissue around the lips (44). In our study, the patient had typical self-mutilative behavior as lip biting, with developmental delay and intellectual disability, but the corresponding *HPRT1* gene variant was not detected by WES. Lesch-Nyhan syndrome is caused by the inactivation or deficiency of hypoxanthine-guanine phosphoribosyl-transferase (HGPRT), while JBTS is a cilia-related disease, which has not been previously associated with HGPRT abnormalities; hence, we suggested that both diseases occurred in one patient. Seizures were the less common clinical feature in JBTS, which has been reported in a few cases (45). We found three cases with seizures in our study, these cases enriched the clinical phenotypes of JBTS.

The variation rate of *CPLANE1* gene is considered to be 8.90% in pure JBTS (42). Two cases with JBTS from the same family that possessed *AHI1* variations were diagnosed with JBTS type 3. The mode of inheritance was autosomal recessive, and the main symptoms were developmental delay and nystagmus. Recent study found that patients with *AHI1* variant are more likely to be complicated by eye, kidney, and liver involvement (46), and these findings are consistent with those described in our study. The *RPGRIP1l* gene variant was detected in one patient, and this was determined to be JBTS type 7, which followed an autosomal recessive inheritance pattern. This patient presented with global developmental delay, hypotonia, and nystagmus, while these findings were consistent with previously published reports (47). *CEP120* variant was detected in one child, and this was determined to be JBTS type 31, which followed an autosomal recessive inheritance pattern. The protein encoded by this gene is known to play a coupling role in the nucleus and centrosome-dependent microtubules, and the symptoms of its dysfunction include nystagmus, strabismus, and apraxia of eye movement (48), which are consistent with our research. We investigated the disease severity and clinical phenotype/genotype of patients underwent WES, and found that JBTS caused by *AHI1* and *CEP120* gene variants were rarely accompanied by extra-neurological abnormalities, and the prognosis after regular rehabilitation treatment was better than others. Studies between clinical phenotypes/genotypes and disease severity have rarely been found yet, considering the different time periods from onset to treatment, the poor adherence in genetic testing, and the sample size of the JBTS patients.

The case of *CHD7* variant, which followed an autosomal dominant inheritance pattern, was diagnosed with CHARGE syndrome combined with typical JBTS manifestations of cerebellar vermis agenesis (Figure 1E). The variation of *CHD7* was on c.4015 C > T (p. Arg1339*), and variant loci for *CHD7* have been reported previously. There are some similar clinical phenotypes between them, such as developmental delay, abnormal eye development, and facial abnormalities (13, 49, 50). Thus far, only two cases of CHARGE syndrome combined with JBTS caused by *CHD7* variant have been reported (14, 15). The WES of the patient reported in the 1990s was not performed (chromosomes were normal), while the other one was found to harbor *de novo* heterozygous variants of *CHD7* gene, namely c.4015C > T (exon 17) (Figure 2A). Furthermore, this variation was identified as pathogenic according to the ACMG guidelines. Therefore, it was speculated that *CHD7* might be related to JBTS, and this finding will enrich the genotypes of JBTS. In the future, we will further verify the functionality of *CHD7* genes to determine whether they are the new genes associated with JBTS. In this study, the remaining patients refused to be examined due to family reasons. We will expand our sample size and provide further enrichment relating to the genetics of JBTS.

Seizures have been observed in more than 10% of JBTS individuals, but no seizure type nor genetic cause appeared to be prevalent (51). Previous reports suggested that JBST with mutations in *CEP290*, *CC2D2A*, *KIAA0586*, *MKS1*, and *TMEM67* present with seizures (30). Three cases in our study had seizures, and it was similar to previously reported findings. In addition, one of the three patients underwent WES, which detected the *CPLANE1* variation, which has not been previously reported. This finding also further enriches the genetic profile of JBTS combined with seizures. However, due to the lack of previous studies, we could not investigate the VEEG characteristics of JBTS patients by performing a literature review. In this study, a total of 26 patients underwent VEEG. Two patients with epilepsy had *abnormal VEEG* showing diffuse slow waves with overlapping sharp waves during wakefulness and sleep. At the first follow-up, one patient died, and another child had developmental regression and poor response to anti-seizure medications. VEEG of the remaining 24 cases was normal. The ratio of the abnormal VEEG in JBTS without epilepsy was low, and the abnormal VEEG was obvious in children with epilepsy, while all patients with abnormal VEEG had JBTS with multi-organ system involvement. Our study provides enrichment for the understanding of VEEG characteristics in children with JBTS and suggests that epilepsy in children with JBTS is difficult to control.

Currently, there is no specific treatment for JBTS. The main treatment plan to improve the child's quality of life is regular rehabilitation and symptomatic treatment to improve the function of the retina, kidneys, liver, and other affected organs. Although exon hopping and other gene-level therapies are under development, there is still a significant issue with JBTS treatment (52, 53). Therefore, follow-up is of great significance if we are to fully evaluate the efficacy and outcomes of the disease. Because previous studies on JBTS have rarely followed up patients in long term, we investigated 36 patients for 34.87 ± 14.33 months and found that the prognosis of children with purely neurological JBTS was better than that of other types of JBTS after rehabilitation treatment. However, the sample size of this study was relatively small, even though it is the largest sample in China. Moreover, multi-center and large-sample clinical studies on this disease are expected in the future.

## 5. Conclusion

We reviewed the clinical phenotypes and partial gene analysis of 36 children with JBTS in our center. Our analysis indicated that almost all children had different degrees of developmental delay. Ocular involvement was the second most common after central nervous system abnormalities combined. Patients with JBTS may have coexisting skin abnormalities. Early MRI examination is helpful for etiological diagnosis. JBTS is a polygenic genetic disease with a poor prognosis, and the *CHD7* gene might be related to JBTS. Regular rehabilitation is significant for children with pure JBTS.

## Data Availability

The original contributions presented in the study are publicly available. This data can be found here: https://www.ncbi.nlm.nih.gov/clinvar/ accession numbers: SCV003802992, SCV003802998, SCV003802999, SCV003803000, SCV003803001, SCV003803014, SCV003803082, SCV003803083, SCV003803084, SCV003803085, SCV003803086, SCV003803087. According to national legislation/guidelines, specifically the Administrative Regulations of the People's Republic of China on Human Genetic Resources (http://www.gov.cn/zhengce/content/2019-06/10/content_5398829.htm, http://english.www.gov.cn/policies/latest_releases/2019/06/10/content_281476708945462.htm), no further datasets presented in this article are readily available. The datasets are available at https://ngdc.cncb.ac.cn/gsa-human/browse/HRA003961 and https://ngdc.cncb.ac.cn/gsa-human/browse/HRA004569. Requests to access the datasets should be directed to the corresponding author.
