# Supplementary material for: Clinical and genetic characteristics of 36 children with Joubert syndrome
Source: Front Pediatr. 2023 Jul 21;11:1102639. doi: 10.3389/fped.2023.1102639 (PMC10401045; doi:10.3389/fped.2023.1102639)
Supplement: Supplementary file 1 [file Table1.docx]

Supplementary Material

Clinical and Genetic Characteristics of 36 Children with Joubert Syndrome

Yan Dong^1, 2*^, Ke Zhang^1, 3^, He Yao^1, 3^, Tianming Jia^1^, Jun Wang^4^, Dengna Zhu^4^, Falin Xu^1^, Meiying Cheng^5^, Shichao Zhao^1^, Xiaoyi Shi^6^

*** Correspondence:** Yan Dong: [yjs6690@126.com](mailto:yjs6690@126.com)

**Supplementary Table**

**Table 1.** Clinical data of patients

|  | **Sex** | **AAD** | **CCM** | **EC** | **AFF** | **P** | **OD** | **HA** | **ARR** | **MT** | **HD** | **LD** | **RI** | **SA** | **WES** | **MRI** | **VEEG** | **ROF** |
| --- | --- | --- | --- | --- | --- | --- | --- | --- | --- | --- | --- | --- | --- | --- | --- | --- | --- | --- |
| 1 | F | 22m | GDR | - | - | - | binocular strabismus | - | - | L | - | - | - | - | NA | MTS  BWS | - | Effective |
| 2 | M | 26m | GDR | - | - | - | - | auditory pathway abnormalities | - | - | - | NA | parenchymal echo enhancement | - | NA | MTS | NA | Developmental regression |
| 3 | M | 11m 19d | GDR | - | shallow nasolabial fold | right thumb polydactyly | - | auditory pathway abnormalities | RDS | L | atrial septal defect | - | - | - | NA | BWS | - | No significant change |
| 4 | F | 38m | GDR | - | - | - | binocular strabismus | - | - | L | - | NA | NA | allergy infection | NA | MTS  (CT) | NA | No significant change |
| 5 | M | 12m | GDR | - | - | - | - | - | - | - | - | - | - | allergy infection | NA | MTS  BWS | NA | Effective |
| 6 | M | 5d | GDR | TCS | - | - | retinal abnormalities | - | RDS | L | atrial septal defect, (reduced left ventricular systolic function) | - | left renal collection  systemic separation | - | NA | MTS  BWS | diffuse slow waves with overlapping sharp waves during wakefulness and sleep | Death |
| 7 | M | 18m | GDR | - | - | - | - | - | - | L | - | NA | NA | - | NA | MTS  BWS | NA | No significant change |
| 8 | M | 2m17d | GDR | - | - | - | nystagmus | - | - | L | - | abnormal liver enzymes | abnormal renal function | - | + | MTS | - | Effective |
| 9 | F | 4m | GDR | - | - | - | optic nerve abnormalities, bilateral optic disc atrophy | cochlear dysplasia and bilateral cystic vestibular semi-circular canal malformation | - | H | increased myocardial enzyme index | abnormal liver enzymes | abnormal renal function | - | + | HOTCV BWS | - | No significant change |
| 10 | F | 2m | GDR | - | - | - | - | - | RDS | - | - | abnormal liver enzymes | - | - | NA | MTS  BWS | NA | No significant change |
| 11 | M | 7m | GDR | - | - | - | - | - | - | - | - | - | abnormal renal function | - | NA | MTS  BWS | - | Effective |
| 12 | M | 36m | GDR | - | - | - | - | - | - | L | - | NA | NA | - | NA | MTS  BWS | NA | Effective |
| 13 | F | 4m | GDR | - | - | - | - | - | - | L | - | NA | NA | - | NA | MTS | NA | Effective |
| 14 | F | 9m | GDR | - | - | - | - | - | - | L | - | - | - | - | + | MTS  BWS | - | Effective |
| 15 | F | 10m | GDR | TCS LNS | low and flat nasal bridge | - | - | - | - | L | - | enlarged liver and spleen | - | - | + | MTS  BWS | diffuse slow waves with overlapping sharp waves during wakefulness and sleep | No significant change, still having seizures |
|  | **Sex** | **AAD** | **CCM** | **EC** | **AFF** | **P** | **OD** | **HA** | **ARR** | **MT** | **HD** | **LD** | **RI** | **SA** | **WES** | **MRI** | **VEEG** | **ROF** |
| 16 | F | 11y | dizziness | - | - | - | - | - | - | - | increased myocardial enzyme  index | NA | NA | - | NA | MTS  BWS | NA | Effective |
| 17 | M | 26m | GDR | - | - | - | - | - | - | L | - | - | - | - | + | MTS | NA | Effective |
| 18 | M | 16m | GDR | - | - | - | - | - | - | - | - | - | - | - | - | MTS  BWS | - | Effective |
| 19 | M | 9m | GDR | - | - | - | binocular strabismus, visual  pathway abnormalities | auditory pathway abnormalities | - | H | - | - | - | - | NA | MTS  BWS | NA | No significant change |
| 20 | F | 12m | OD | - | - | - | binocular strabismus | - | - | - | - | NA | NA | - | NA | MTS | NA | No significant change |
| 21 | M | 12m | GDR | - | - | - | visual  pathway abnormalities | - | - | - | - | - | - | - | NA | MTS  BWS | - | Effective |
| 22 | M | 8m | GDR | - | - | - | nystagmus | - | - | L | - | - | - | - | + | BWS | - | Effective |
| 23 | M | 8m | GDR | - | - | - | nystagmus | - | - | L | - | - | - | - | + | MTS  BWS | - | Effective |
| 24 | F | 6m | GDR | - | - | - | nystagmus | - | - | - | - | - | parenchymal echo enhancement | rash | NA | MTS  BWS | - | Developmental regression |
| 25 | M | 5m | GDR | - | - | right toe polydactyly | binocular strabismus | auditory pathway abnormalities | - | - | increased myocardial enzyme  index | - | - | rash | NA | MTS  BWS | - | No significant change |
| 26 | F | 5m | GDR | - | - | - | - | - | RDS | - | - | - | - | - | NA | MTS  BWS | - | Death |
| 27 | F | 27m | GDR | - | - | - | - | - | - | L | - | - | - | - | NA | MTS | - | Effective |
| 28 | F | 3m | GDR | - | - | - | nystagmus, optic nerve abnormalities | - | RDS | - | - | - | - | - | NA | MTS  BWS | - | No significant change |
| 29 | F | 10m | GDR | - | - | - | - | auditory pathway abnormalities | - | L | - | NA | NA | - | + | MTS | - | Effective |
| 30 | F | 12m | GDR | - | - | - | - | auditory pathway abnormalities | - | - | - | - | - | - | NA | MTS | - | Effective |
| 31 | F | 6m | GDR | - | - | - | - | - | - | L | - | - | - | - | NA | MTS  BWS | NA | Effective |
| 32 | M | 3m | GDR | - | - | - | - | - | - | L | - | - | - | - | NA | MTS  BWS | NA | Effective |
| 33 | F | 5m | GDR | - | wide eye distance | - | - | - | neonatal intermit-ent apnoea | L | - | - | - | - | NA | MTS  BWS | NA | No significant change |
| 34 | F | 24m | MDR | - | - | - | - | - | - | - | - | - | - | rash | NA | MTS  BWS | NA | No significant change |
|  | **Sex** | **AAD** | **CCM** | **EC** | **AFF** | **P** | **OD** | **HA** | **ARR** | **MT** | **HD** | **LD** | **RI** | **SA** | **WES** | **MRI** | **VEEG** | **ROF** |
| 35 | M | 6m15d | GDR | BHS | - | - | nystagmus, binocular strabismus, retinal abnormalities | - | - | L | - | - | - | - | NA | BWS | - | Effective |
| 36 | M | 10d | GDR | - | - | - | retinal abnormalities | auditory pathway abnormalities | RDS | - | - | NA | abnormal renal function | - | NA | BWS | NA | Effective |

**Abbreviations**: AAD, age at diagnosis; AFF, abnormal facial features; ARR, abnormal respiratory rhythm; BHS, breath-holding spells; BWS, bat wing sign; CCM, core clinical manifestations; CT, computed tomography; EC, epileptic complications; F, female; GDR, global developmental retardation; H: high; HA, hearing abnormalities; HD, heart diseases; HOTCV, hypoplasia of the cerebellar vermis; L, low; LD, liver damage; LNS, lesch-nyhan syndrome; M, male; MDR, motor developmental retardation; MRI, magnetic resonance imaging; MT, muscle tension; MTS, molar tooth sign; NA, not available; NRDS, neonatal respiratory distress syndrome; OD, ocular diseases; OM, occipital meningocele; P, polydactylism; RI, renal injury; ROF, result of follow-up; SA, skin abnormalities; TCS, tonic-clonic seizures; VEEG, video electroencephalogram; WES, whole exon sequencing; +, abnormal; -, normal
